# Evaluation of a Point-of-Care Test for Pre-Vaccination Testing to Detect Antibodies against Canine Adenoviruses in Dogs

**DOI:** 10.3390/v13020183

**Published:** 2021-01-26

**Authors:** Michèle Bergmann, Mike Holzheu, Yury Zablotski, Stephanie Speck, Uwe Truyen, Katrin Hartmann

**Affiliations:** 1 Clinic of Small Animal Medicine, Centre for Clinical Veterinary Medicine, LMU Munich, Veterinaerstrasse 13, 80539 Munich, Germany; Y.Zablotski@med.vetmed.uni-muenchen.de (Y.Z.); hartmann@lmu.de (K.H.); 2 Clinic of Small Animal Surgery and Reproduction, Centre for Clinical Veterinary Medicine, LMU Munich, Veterinaerstrasse 13, 80539 Munich, Germany; M.Holzheu@medizinische-kleintierklinik.de; 3 Institute of Animal Hygiene and Veterinary Public Health, University of Leipzig, An den Tierkliniken 1, 04103 Leipzig, Germany; stephanie@speck-kaysser.de (S.S.); truyen@vetmed.uni-leipzig.de (U.T.)

**Keywords:** antibodies, CAV, CAV-1, CAV-2, in-house, protection, vaccination, sensitivity, specificity, PPV, NPV

## Abstract

(1) Background: Antibody testing is commonly used to assess a dog’s immune status. For detection of antibodies against canine adenoviruses (CAVs), one point-of-care (POC) test is available. This study assessed the POC test´s performance. (2) Methods: Sera of 198 privately owned dogs and 40 specific pathogen-free (SPF) dogs were included. The reference standard for detection of anti-CAV antibodies was virus neutralization (VN) using CAV-1 and CAV-2 antigens. Specificity, sensitivity, positive predictive value (PPV), negative predictive value (NPV), and overall accuracy (OA) of the POC test were assessed. Specificity was considered most important. (3) Results: Prevalence of CAV-1 neutralizing antibodies (≥10) was 76% (182/238) in all dogs, 92% (182/198) in the subgroup of privately owned dogs, and 0% (0/40) in SPF dogs. Prevalence of CAV-2 neutralizing antibodies (≥10) was 76% (181/238) in all dogs, 91% (181/198) in privately owned dogs, and 0% (0/40) in SPF dogs. Specificity for detection of CAV-1 antibodies was lower (overall dogs, 88%; privately owned dogs, 56%; SPF dogs, 100%) compared with specificity for detection of CAV-2 antibodies (overall dogs, 90%; privately owned dogs, 65%; SPF dogs, 100%). (4) Conclusions: Since false positive results will lead to potentially unprotected dogs not being vaccinated, specificity should be improved to reliably detect anti-CAV antibodies that prevent infectious canine hepatitis in dogs.

## 1. Introduction

Two adenoviruses are important pathogens in dogs. Canine adenovirus-1 (CAV-1) can cause a severe systemic disease, the infectious canine hepatitis (ICH), which affects the liver (necrohemorrhagic hepatitis), kidneys (interstitial nephritis), and/or eyes (uveitis, corneal edema) [1,2,3]. Thus, every dog should be protected against ICH, at least in regions where CAV-1 is still present [4,5]. Another closely related adenovirus affecting dogs is canine adenovirus-2 (CAV-2), which is one of the numerous pathogens able to cause canine infectious respiratory disease (CIRD) [6]. Since vaccination with modified live CAV-1 has induced severe adverse events in the past [7], dogs are now regularly vaccinated with modified live CAV-2 [8]. Due to their close antigenic relationship, vaccination against CAV-2 or infection with CAV-2 also induces immunity against CAV-1 [9,10].

Detection of anti-CAV antibodies in adult dogs correlates with protection independent of the antibody titer, because antibodies at any level indicate the presence of immunological memory cells that are able to rapidly produce (even more) antibodies in the event of infection [11,12]. Therefore, antibody testing presents the ideal possibility to evaluate the specific immunity of dogs to CAV-1 and CAV-2. This tool can be used before vaccination and thus is useful in avoiding unnecessary vaccinations in dogs that already have antibodies and are therefore protected; re-vaccination in these dogs likely has no beneficial effect, since pre-vaccination antibodies can prevent modified live virus from replication, resulting in lack of immune response [5]. In addition, antibody testing can be used during the management of disease outbreaks [13].

Virus neutralization (VN) is the reference standard for measurement of antibodies against CAV; however, results are not immediately available, since serum samples have to be sent to a diagnostic laboratory. Reliable point-of-care (POC) tests that rapidly determine whether a dog has antibodies or not during a health care appointment at a veterinary practice would be useful in deciding whether a dog should be vaccinated or not.

For the detection of anti-CAV antibodies in practice, one POC test, the ImmunoComb^®^ Canine VacciCheck (Biogal Laboratories), an enzyme-linked immunosorbent assay (ELISA), is commercially available. The performance of the POC test to determine anti-CAV antibodies has never been evaluated in independent studies.

The present study assessed the specificity, sensitivity, positive predictive value (PPV), negative predictive value (NPV), and overall accuracy (OA) in the detection of anti-CAV antibodies of a commercially available POC test, in comparison to the reference standard VN’s detection of CAV-1 and CAV-2 antibodies.

## 2. Materials and Methods

### 2.1. Sera

The study included privately owned dogs that were brought to the Clinic of Small Animal Medicine (LMU Munich) between June and August 2018, and from which blood was drawn for any reason (n = 198). The study was authorized by the ethical committee of the Centre for Veterinary Clinical Medicine, LMU Munich, Germany (license number 124-13-05-2018).

The dogs’ ages ranged from 3 months to 16 years; median age was 9 years. The dogs belonged to a variety of different breeds (n = 151) or were mixed-breed (n = 47), were male (n = 96) or female (n = 102), and intact (n = 97) or neutered (n = 101). Vaccination status was correct (n = 104), incomplete (n = 28), or unknown (n = 66). When presented to the clinic, dogs were healthy (n = 22) or had various diseases (n = 176) (see also Table S1).

Additionally, the study included 40 serum samples of specific pathogen-free (SPF) dogs that had never received a vaccination. The additional samples were provided by the Institute of Animal Hygiene and Veterinary Public Health, University of Leipzig. All samples were stored at −80 °C.

### 2.2. Measurement of Antibodies against CAV-1 and CAV-2 by VN

All serum samples were analyzed in duplicate by VN in two different assays, one using a CAV-1 strain and the other one using a CAV-2 strain. For both assays, heat-treatment of samples was performed at 56 °C for 30 min. One hundred microliters of each serum was diluted in phosphate-buffered saline (pH 7.2); the first dilution step was 1:5. Afterwards, samples were further diluted at steps 1:2. Then, all dilutions were mixed with an equal volume of CAV-1 isolate Ag219 or CAV-2 isolate DU18 (200 median tissue culture infective dose/0.1 mL) and incubated (37 °C, 60 min). Madin–Darby Canine Kidney (MDCK) cells were maintained in Dulbecco’s Modified Eagle’s Medium (Merck Millipore, Darmstadt, Germany) supplemented with 5% fetal calf serum (FCS; Merck Millipore), 1% nonessential amino acids (Merck Millipore), and 1% Penicillin–Streptomycin (Merck Millipore) at 37 °C, 5% CO_2_. Subsequently, MDCK cells seeded in 96-well microtiter plates were inoculated with 100 μL of these serum/virus mixtures. Plates were incubated for 5 days at 37 °C, 5% CO_2_. Microtiter plates were examined daily using an inverted microscope at a 10× magnification. The presence of cytopathic effects indicated viral spread within the cell monolayer. The positive control serum was provided by the Baker Institute for Animal Health, Cornell University, Ithaca, NY, USA. A VN antibody titer upon the first dilution was considered positive (≥10).

### 2.3. Measurement of Antibodies against CAV by POC Test

All serum samples were analyzed with the ImmunoComb^®^, a POC test based on an ELISA principle to detect antibodies against CAV, canine parvovirus (CPV), and canine distemper virus (CDV). Data on antibodies against CPV and CDV were not the subject of this study. The POC test kit was stored refrigerated and acclimatized to room temperature for 60 min before testing. Testing was performed using serum (5 µL), following the multi-stage step-test protocol of the manufacturer by an independent person blinded to the VN results. The manufacturer declares an anti-adenovirus cut-off titer of ≥16 as positive.

### 2.4. Data Analyses

Results of the POC tests were compared to the reference VN assay using CAV-1 and CAV-2 to determine the diagnostic performance of the POC test. R version 3.6.3 (2020-02-29) (R Foundation for Statistical Computing, Vienna, Austria) was used to calculate sensitivity (rate of true positives), specificity (rate of true negatives), positive predictive value (PPV; proportion of dogs with positive test results in total of the dogs within a population with positive results), negative predictive value (NPV; proportion of dogs with negative test results in total of the dogs within a population with negative results), and overall accuracy (OA; number of correctly predicted POC test results) for all dogs and for both subgroups (privately owned dogs and SPF dogs); 95% confidence intervals were calculated to estimate uncertainties. Specificity was regarded as the most important performance parameter, since false-positive results can result in potentially unprotected dogs not being vaccinated.

## 3. Results

### 3.1. Prevalence of Neutralizing Antibodies against CAV-1 and CAV-2 Determined by VN

The prevalence of neutralizing antibodies against CAV-1, when considering a VN titer cut-off point of ≥10 as positive, was 76% (182/238; CI_95%_: 71–82) in all dogs, 92% (182/198; CI_95%_: 87–95) in privately owned dogs, and 0% (0/40) in SPF dogs (Table 1). Antibody titers ranged from 10 to 2560 (Table 2). The prevalence of neutralizing antibodies against CAV-2, when considering a VN titer cut-off point of ≥10 as positive, was 76% (181/238; CI_95%_: 71–82) in all dogs, 91% (181/198; CI_95%_: 87–95) in privately owned dogs, and 0% (0/40) in SPF dogs (Table 1). Antibody titers ranged from 10 to 1280 (Table 2). All duplicate tests revealed the same VN test result.

Overall, 97% of dogs with neutralizing antibodies against CAV-1 (176/182; CI_95%_: 93–99) also had CAV-2-neutralizing antibodies. Six dogs were anti-CAV-1 antibody-positive (titer range: 10–80; median titer: 20) but anti-CAV-2 antibody-negative (Table 3). Five dogs were anti-CAV-1 antibody-negative but anti-CAV-2 antibody-positive (titer range: 10–40; median titer: 40). In total, anti-CAV-1 and anti-CAV-2 antibody titers differed (≥2 titer steps) in 18% (43/238) of the dogs.

### 3.2. Measurement of Antibodies against CAV by POC Test

The multi-stage step testing procedure of the POC test was considered relatively labor-intensive. All results of the POC tests could clearly be classified as positive or negative. In comparison to VN using CAV-1, the POC test delivered 85 false-negative and 7 false-positive results (Table 1); the POC test´s specificity, sensitivity, PPV, NPV, and OA in all dogs was 88%, 53%, 93%, 37%, and 61%, respectively, and in privately owned dogs, 56%, 53%, 93%, 10%, and 54%, respectively. When evaluating only sera of SPF dogs, the specificity of the POC test was 100% (Table 4). In comparison to VN using CAV-2, the POC test delivered 82 false-negative and 6 false-positive results (Table 2); the POC test´s specificity, sensitivity, PPV, NPV, and OA in all dogs was 90%, 55%, 94%, 38%, and 63%, respectively, and in privately owned dogs, 65%, 55%, 94%, 12%, and 55%, respectively. When evaluating only sera of SPF dogs, the specificity of the POC test was 100% (Table 4).

## 4. Discussion

ICH has become a rare disease in Europe. Nevertheless, over the last few decades, outbreaks have repeatedly been observed, mainly in shelter dogs in Italy [1,14] but also in Switzerland [3]. In Germany, only one case, a dog imported from Spain, has been described during the last 20 years [15]. However, the presence of CAV-1 in a client-owned dog population in Italy confirms the continuous risk of infection, at least in Southern Europe; a recent Italian study included 51 dogs that were presented to a veterinary clinic in Bologna for reasons unrelated to CAV-infection; 4 of these dogs (8%) were PCR-positive for CAV-1 and 30 dogs (59%) were PCR-positive for CAV-2. Results of a sequence analysis indicated that a genetically stable CAV-1 strain and different CAV-2 strains circulated in this population [16]. The export of indigenous dogs to surrounding countries, as well as travelling, might allow CAV-1 (as well as CAV-2) to re-spread to Central and Northern Europe. In unprotected dogs, ICH is a disease with a high mortality rate [3]. In Italy, however, infected dogs only showed mild clinical signs, likely because they had been vaccinated in the past [16]. Thus, dogs should be reliably protected [5], at least when they come from or travel to regions in which cases of ICH have been observed during the last decades (e.g., Italy [1,14,16]).

Experimental vaccination studies have shown the long-term presence of anti-CAV antibodies that correlate with protection in previously vaccinated adult dogs [5,10,12] for up to 9 years after vaccination [17]. In field studies, antibodies were present after 6 to 14 years [17,18]. Individual dogs, however, were more likely to lack antibodies if the last vaccination was given >3 [19] and ≥4 years [20] ago. Currently, guidelines recommend 3-yearly boosters for vaccination against ICH [4,5]. Nevertheless, since no vaccine is risk-free for the individual dog, antibody testing before vaccination would be preferable, as many dogs are likely still protected 3 years after vaccination [5].

In addition, antibody testing can be useful to evaluate the dog’s vaccination response; the absence of anti-CAV antibodies in dogs after vaccination indicates a non-response, (e.g., due to interference with maternally derived antibodies, pre-existing antibodies due to previous infection or vaccination, or immunodeficiency [19]). Further, antibody testing is helpful for the separation of dogs during the management of disease outbreaks. Therefore, availability of reliable antibody tests is desirable.

Anti-CAV antibodies can be determined either in a diagnostic laboratory using the reference standard VN or in a veterinary practice by a POC test. The latter is more helpful, e.g., when creating an individual vaccination scheme during the health care appointment. Only one POC test, the ImmunoComb^®^, that detects antibodies against CAV is commercially available today, but it has so far not been evaluated by independent studies.

In the present study, reference standard VN was performed using two different strains (CAV-1 and CAV-2). This comparison was of particular interest, since the isolates with which dogs had previously been in contact were unknown. Furthermore, the POC test’s manufacturer gives no information on which strains are detected by the POC test. It is generally believed that anti-CAV antibodies cross-protect against CAV-1 and CAV-2; this, however, seems not to be guaranteed. A study in Northern Italy found a relatively high number of dogs (12%; 6/51) that were coinfected, 4 of them with CAV-1 and CAV-2. Two other dogs were coinfected with 2 different CAV-2 strains, indicating that a first immune response did not necessarily protect against another CAV infection [16].

In the present study, most dogs (97%) had antibodies with CAV-1 and CAV-2 neutralizing properties, leading to the conclusion that dogs are commonly protected against both, ICH and CIRD-associated CAV-2 infection. However, 5 dogs had antibodies only neutralizing CAV-2 but not CAV-1, and 6 dogs had antibodies only neutralizing CAV-1 but not CAV-2. This is an interesting finding and could indicate that cross-protection is not always present, and that complex immune reactions in individual dogs might differ. Generally, however, there was a large agreement in antibody results against CAV-1 and CAV-2, indicating that CAV-2 is useful as vaccine antigen to protect against CAV-1.

The specificities of the POC test in the present study, especially when considering VN results using CAV-1 (all dogs, 88%; privately owned dogs, 56%), were lower than those reported by the manufacturer (93%), who also used VN as the reference standard with a cut off ≥16 (anti-CAV antibody prevalence: 66%) [21]. The lower specificity is especially of concern because, with an increasing number of false positive results, more dogs would not receive a vaccination even though they are potentially not protected. In these cases, individual dogs might be susceptible to ICH. False-positive results could even lead to a decrease in herd immunity, and thus to a higher risk of ICH outbreaks within a dog population.

The PPV predicts the probability that a dog that was tested antibody-positive is correctly classified as having antibodies. In the present study, the PPV was high (93%); this means that POC test-positive dogs are truly positive in 93% of the cases. However, the PPV is prevalence-dependent and decreases with decreasing antibody prevalence. Thus, the high PPV depended on the high antibody prevalence; it was nearly identical to the anti-CAV-1 antibody prevalence (92%) in the present study.

The sensitivities of the POC test in the present study (53% in comparison to VN using CAV-1; 55% in comparison to VN using CAV-2) were much lower compared to those determined by the manufacturer (94%). Many dogs had a false-negative test result. Although this might not be such a big concern given that 3-yearly boosters are currently performed (unnecessarily) in many dogs, it also limits the usefulness of the test.

The reason for the different results in the present study in comparison with the manufacturer’s reports could be caused by the use of different batches or adjustments of the POC test by the manufacturer. Incorrect VN results are less likely, since all duplicate tests revealed the same respective titer. Differences in specificity might be due to the higher prevalence of anti-CAV antibodies in the present study (overall dogs, 76%) in comparison with the ones in the manufacturer’s study (66%). However, former studies found an even higher prevalence of antibodies against CAV of 96% [20,22]. The goal of a POC test should be to precisely identify field dogs lacking antibodies and, therefore, are unprotected (e.g., immunocompromised dogs). For this reason, and in order to prevent occurrence of false-positive results, modifications of the POC test to increase specificity are recommended. A limitation of the study is that time since last vaccination was not recorded in most of the privately owned dogs, which would have provided more insights into the specific immune status.

## 5. Conclusions

The POC test did not perform well enough to be recommended for use in private practice. Since false-positive results will lead to potentially unprotected dogs not being vaccinated, modifications of the POC test are necessary to improve its specificity for the detection of CAV-1 antibodies.

## Figures and Tables

**Table 1:** Point-of-care test results to detect anti-canine adenovirus antibodies in sera of all 238 dogs (198 privately owned dogs and 40 specific pathogen-free dogs) and comparison to virus neutralization against canine adenovirus-1 and against canine adenovirus-2 as the reference standard.

VN ^1^ Result against Canine Adenovirus-1	POC Test ^2^ Result	
	Negative	Positive
**Results of all 238 dogs (privately owned dogs and specific pathogen-free dogs)**		
negative (n = 56)	49 true negatives	7 false positives
positive (n = 182)	85 false negatives	97 true positives
**Results of 198 privately owned dogs**		
negative (n = 16)	9 true negatives	7 false positives
positive (n = 182)	85 false negatives	97 true positives
**Results of 40 specific pathogen-free dogs**		
negative (n = 40)	40 true negatives	0 false positives
positive (n = 0)	0 false negatives	0 true positives
**VN ^1^ Result against Canine Adenovirus-2**	**POC test ^2^ result**	
	**Negative**	**Positive**
**Results of all 238 dogs (privately owned dogs and specific pathogen-free dogs)**		
negative (n = 57)	51 true negatives	6 false positives
positive (n = 181)	82 false negatives	99 true positives
**Results of 198 privately owned dogs**		
negative (n = 17)	11 true negatives	6 false positives
positive (n = 181)	82 false negatives	99 true positives
**Results of 40 specific pathogen-free dogs**		
negative (n = 40)	40 true negatives	0 false positives
positive (n = 0)	0 false negatives	0 true positives

^1^ VN, virus neutralization; ^2^ POC test, point-of-care test.

**Table 2:** Point-of-care test results and number of dogs with the respective antibody titers in virus neutralization against canine adenovirus-1 and canine adenovirus-2.

POC ^2^ Test Results	VN ^1^ Results against Canine Adenovirus-1 and the Respective Antibody Titers									
	(<10)	10	20	40	80	160	320	640	1280	2560
positive	7	3	8	12	14	20	15	18	3	4
negative	49	4	7	17	15	30	10	1	1	0
total	56	7	15	29	29	50	25	19	4	4
**POC ^2^ Test Results**	**VN ^1^ Results against Canine Adenovirus-2 and the Respective Antibody Titers**									
	(<10)	10	20	40	80	160	320	640	1280	2560
positive	6	3	9	14	21	29	8	10	5	0
negative	51	7	8	23	26	14	4	0	0	0
total	57	10	17	37	47	43	12	10	5	0

^1^ VN, virus neutralization; ^2^ POC test, point-of-care test.

**Table 3:** Cross-classified virus neutralization test results of 11 dogs that were anti-canine adenovirus- (CAV-)2 antibody-positive but anti-CAV-1 antibody-negative (<10) or anti-CAV-1 antibody-positive but anti-CAV-2 antibody-negative (<10).

Dog	Anti-CAV ^1^ -1 Antibody Titer	Anti-CAV ^1^ -2 Antibody Titer	POC Test ^2^ Result
1	<10	10	negative
2	<10	10	negative
3	<10	40	negative
4	<10	40	positive
5	<10	40	positive
6	10	<10	negative
7	20	<10	negative
8	20	<10	negative
9	20	<10	positive
10	40	<10	negative
11	80	<10	negative

^1^ CAV, canine adenovirus; ^2^ POC test, point-of-care test.

**Table 4:** Performance parameters of the point-of-care test to detect canine-adenovirus-neutralizing antibodies based on the results in Table 1; sensitivity, specificity, positive predictive value, negative predictive value, and overall accuracy were computed using virus neutralization against canine adenovirus-1 and against canine adenovirus-2 as the reference standard.

Antibody Prevalence in VN ^1^ against Canine Adenovirus-1% (CI ^2^_95%_ %)	Sensitivity % (CI ^2^_95%_ %)	Specificity % (CI ^2^_95%_ %)	PPV ^3^ % (CI ^2^_95%_ %)	NPV ^4^ % (CI ^2^_95%_ %)	OA ^5^ % (CI ^2^_95%_ %)
**Results of all 238 dogs (privately owned dogs and specific pathogen-free dogs)**					
76 (71–82)	53 (46–61)	88 (76–95)	93 (87–97)	37 (28–45)	61 (55–68)
**Results of 198 privately owned dogs**					
92 (87–95)	53 (45–60)	56 (30–80)	93 (87–97)	10 (4–17)	54 (46–61)
**Results of 40 specific pathogen-free dogs**					
0	n. d. ^6^	100	n. d. ^6^	100	100
**Antibody prevalence in VN ^1^ against canine adenovirus-2 % (CI ^2^_95%_ %)**	**Sensitivity % (CI ^2^_95%_ %)**	**Specificity % (CI ^2^_95%_ %)**	**PPV ^3^ % (CI ^2^_95%_ %)**	**NPV ^4^ % (CI ^2^_95%_ %)**	**OA ^5^ % (CI ^2^_95%_ %)**
**Results of all 238 dogs (privately owned dogs and specific pathogen-free dogs)**					
76 (71–82)	55 (47–62)	90 (79–95)	94 (86–96)	38 (29–45)	63 (57–69)
**Results of 198 privately owned dogs**					
91 (87–95)	55 (47–62)	65 (41–83)	94 (86–96)	12 (6–19)	55 (49–62)
**Results of 40 specific pathogen-free dogs**					
0	n. d. ^6^	100	n. d. ^6^	100	100

^1^ VN, virus neutralization (titers ≥10 were considered positive); ^2^ CI, confidence interval; ^3^ PPV, positive predictive value (proportion of dogs with positive test results, in total, of the dogs within a population with positive results); ^4^ NPV, negative predictive value (proportion of dogs with negative test results, in total, of the dogs within a population with negative results); ^5^ OA, overall accuracy (number of correctly predicted POC test results; ^6^ n. d., could not be determined.

## Data Availability

Data is contained within the article or Supplementary Materials).

## Supplementary Materials

The following is available online at https://www.mdpi.com/1999-4915/13/2/183/s1, Table S1: Signalment, vaccination status and health status of the 198 dogs included in the study and anti-canine adenovirus antibody test results of the point-of-care-test and the respective antibody titers in virus neutralization using canine adenovirus-1 and -2.
