# Supplementary material for: Evaluation of a Point-of-Care Test for Pre-Vaccination Testing to Detect Antibodies against Canine Adenoviruses in Dogs
Source: Viruses. 2021 Jan 26;13(2):183. doi: 10.3390/v13020183 (PMC7911502; doi:10.3390/v13020183)
Supplement: Supplementary file 1 [file viruses-13-00183-s001.pdf]

Supplemental File Table 1 Signalment, vaccination status, and health status of the 198 dogs included in the study and anti-canine adenovirus antibody test results of the point-of-care-test and the respective antibody titers in virus neutralization using canine adenovirus-1 and -2.

privately owned dogs	breed	sex	neutering status	age in years	vaccination status	health status	point-of-care test result	antibody titer against CAV[1]-1 in virus neutralization	antibody titer against CAV[1]-2 in virus neutralization
1	mix	female	intact	7	correct vaccinated	immune-mediated disorder	positive	160	80
2	mix	female	neutered	5	unknown	tumour	negative	160	80
3	Bearded Collie	female	intact	12	unknown	endocrine disorder	positive	320	160
4	Labrador	female	intact	11	correct vaccinated	tumour	positive	40	160
5	mix	female	neutered	11	unknown	healthy	positive	320	160
6	mix	female	intact	16	correct vaccinated	tumour	negative	160	80
7	Dachshund	female	intact	7	unknown	inflammatory/infectious disease	negative	320	160
8	French Bulldog	male	intact	3	correct vaccinated	inflammatory/infectious disease	negative	<10	<10
9	Caucasian Shepherd	male	neutered	5	unknown	neurologic disorder	negative	160	80
10	Havanese	male	neutered	5	unknown	inflammatory/infectious disease	negative	80	80
11	Bernedoodle	male	intact	1	correct vaccinated	orthopedic disorder	positive	640	640
12	Golden Retriever	female	neutered	15	unknown	tumour	negative	80	10
13	mix	female	intact	10	unknown	tumour	positive	160	80
14	Labrador	female	neutered	10	unknown	neurologic disorder	positive	160	40
15	French Bulldog	male	intact	8	correct vaccinated	tumour	negative	160	20
16	Jack Russel Terrier	male	intact	15	correct vaccinated	healthy	negative	40	10
17	mix	female	neutered	7	unknown	inflammatory/infectious disease	positive	160	160

18	Golden Retriever	male	intact	5	unknown	tumour	negative	40	10
19	Irish Setter	female	intact	2	unknown	orthopedic disorder	negative	20	10
20	Chihuahua	male	neutered	13	incomplete	cardiac disorder	positive	640	160
21	American Akita	male	intact	5	correct vaccinated	inflammatory/infectious disease	positive	2560	1280
22	Boxer	male	intact	5	incomplete	healthy	positive	160	160
23	Dachshund	male	neutered	7	unknown	various disorder	positive	160	160
24	Jack Russel Terrier	male	neutered	15	unknown	orthopedic disorder	positive	1280	160
25	German Shepherd	male	neutered	4	correct vaccinated	tumour	positive	80	20
26	Dalmatian	male	neutered	5	unknown	orthopedic disorder	positive	80	20
27	Doberman	male	intact	2	correct vaccinated	healthy	negative	160	40
28	mix	female	neutered	15	incomplete	inflammatory/infectious disease	negative	40	20
29	mix	female	neutered	16	unknown	cardiac disorder	positive	80	40
30	mix	female	neutered	15	correct vaccinated	endocrine disorder	negative	160	40
31	Doberman	female	intact	4	correct vaccinated	cardiac disorder	positive	160	160
32	Australian Shepherd	female	intact	1	correct vaccinated	cardiac disorder	negative	<10	<10
33	German Shorthair	male	intact	13	unknown	neurologic disorder	negative	320	80
34	Australian Shepherd	male	neutered	13	unknown	inflammatory/infectious disease	positive	160	320
35	Golden Red Retriever	female	intact	6	unknown	orthopedic disorder	positive	640	640
36	mix	male	neutered	15	unknown	orthopedic disorder	negative	20	40
37	mix	female	neutered	7	incomplete	inflammatory/infectious disease	negative	160	40

38	Boston Terrier	female	neutered	11	correct vaccinated	cardiac disorder	positive	40	20
39	Boxer	female	intact	1	correct vaccinated	inflammatory/infectious disease	negative	20	20
40	Dachshund	male	intact	12	correct vaccinated	cardiac disorder	positive	640	320
41	Samoyed	female	neutered	11	unknown	tumour	negative	160	80
42	Beardie Pon	female	intact	14	correct vaccinated	cardiac disorder	negative	160	80
43	Dachshund	male	intact	11	unknown	endocrine disorder	negative	160	160
44	Labrador	male	neutered	11	unknown	tumour	negative	160	40
45	Beagle	female	neutered	14	unknown	tumour	negative	20	< 10
46	Cocker Spaniel	male	neutered	10	correct vaccinated	tumour	negative	40	40
47	Irish Setter	male	intact	5	correct vaccinated	neurologic disorder	positive	80	80
48	German Shepherd	male	neutered	12	unknown	tumour	positive	2560	1280
49	Dalmatian	male	neutered	13	correct vaccinated	neurologic disorder	negative	320	160
50	mix	male	intact	11	correct vaccinated	tumour	negative	80	40
51	mix	male	neutered	10	unknown	tumour	positive	2560	640
52	Rhodesian Ridgeback	male	intact	13	incomplete	tumour	negative	<10	<10
53	Spitz	male	neutered	10	unknown	inflammatory/infectious disease	negative	40	40
54	French Bulldog	male	intact	3	unknown	various disorder	negative	40	40
55	Doberman	female	neutered	5	unknown	various disorder	negative	80	40
56	Airdale Terrier	male	intact	11	unknown	tumour	negative	160	80
57	Jack Russel Terrier	female	intact	9	unknown	cardiac disorder	negative	10	<10

58	Giant Schnauzer	female	neutered	11	unknown	tumour	positive	640	80
59	Chihuahua	female	intact	3	correct vaccinated	inflammatory/infectious disease	positive	640	640
60	mix	female	neutered	13	unknown	endocrine disorder	negative	80	20
61	American Bulldog	male	neutered	8	correct vaccinated	tumour	negative	320	160
62	Doberman	male	intact	9	unknown	neurologic disorder	positive	160	80
63	Great Dane	female	intact	9	correct vaccinated	tumour	positive	640	320
64	Australian Shepherd	female	neutered	11	unknown	tumour	negative	<10	10
65	mix	female	neutered	12	correct vaccinated	tumour	positive	640	320
66	Tornjak	female	neutered	3	correct vaccinated	various disorder	negative	<10	<10
67	mix	male	intact	7	unknown	orthopedic disorder	negative	160	80
68	mix	female	neutered	4	correct vaccinated	inflammatory/infectious disease	positive	320	160
69	Magyar Vizsla	male	intact	14	unknown	tumour	negative	40	10
70	Labrador	male	neutered	14	correct vaccinated	various disorder	negative	40	<10
71	Akita Inu	male	intact	6	correct vaccinated	various disorder	positive	640	160
72	mix	male	neutered	11	unknown	tumour	negative	160	80
73	mix	male	neutered	9	unknown	healthy	negative	80	40
74	Beauceron	male	neutered	11	correct vaccinated	orthopedic disorder	negative	80	80
75	Shih Tzu	male	intact	5	correct vaccinated	cardiac disorder	negative	320	320
76	Boxer	male	intact	12	incomplete	inflammatory/infectious disease	positive	320	160
77	French Bulldog	male	neutered	7	unknown	tumour	positive	80	80

78	mix	female	neutered	11	unknown	tumour	negative	160	40
79	Pomeranian	female	intact	9	incomplete	orthopedic disorder	positive	160	40
80	Newfoundland	female	neutered	6	correct vaccinated	cardiac disorder	negative	160	80
81	mix	male	neutered	14	correct vaccinated	orthopedic disorder	positive	640	1280
82	Bracco Italiano	male	intact	2	correct vaccinated	inflammatory/infectious disease	negative	<10	10
83	mix	female	neutered	13	incomplete	tumour	negative	80	40
84	Labrador	female	intact	2	correct vaccinated	various disorder	negative	80	40
85	English Bulldog	male	intact	1	correct vaccinated	inflammatory/infectious disease	positive	640	320
86	mix	female	intact	13	correct vaccinated	tumour	positive	2560	640
87	Pug	male	intact	8	unknown	orthopedic disorder	positive	80	80
88	Golden Retriever	female	neutered	13	correct vaccinated	tumour	positive	40	160
89	Podenco Canario	female	neutered	13	unknown	inflammatory/infectious disease	negative	20	<10
90	Pug	female	intact	11	unknown	various disorder	positive	320	160
91	Australian Shepherd	male	intact	2	correct vaccinated	neurologic disorder	negative	160	80
92	mix	female	intact	1	correct vaccinated	inflammatory/infectious disease	positive	160	80
93	Hovawart	female	neutered	9	incomplete	cardiac disorder	positive	160	80
94	Bearded Collie	male	intact	9	correct vaccinated	orthopedic disorder	positive	80	40
95	Pug	male	intact	10	unknown	tumour	positive	320	160
96	Eurasier	female	neutered	7	correct vaccinated	various disorder	negative	40	160
97	German Shepherd	male	intact	3	correct vaccinated	inflammatory/infectious disease	positive	320	160

98	Golden Retriever	male	intact	12	correct vaccinated	neurologic disorder	positive	20	20
99	Jack Russel Terrier	female	neutered	1	correct vaccinated	healthy	positive	160	80
100	Labrador	male	intact	14	correct vaccinated	cardiac disorder	negative	80	<10
101	Miniature Dachshund	female	intact	0.25	incomplete	inflammatory/infectious disease	negative	<10	<10
102	German Shorthair	male	intact	7	unknown	orthopedic disorder	negative	80	160
103	mix	female	intact	13	correct vaccinated	tumour	positive	40	20
104	Beagle	male	intact	1	incomplete	inflammatory/infectious disease	negative	160	80
105	mix	female	intact	16	correct vaccinated	neurologic disorder	positive	10	20
106	Weimaraner	male	intact	3	correct vaccinated	neurologic disorder	negative	160	160
107	Labradoodle	female	intact	7	incomplete	various disorder	negative	160	160
108	German Shorthair	female	neutered	3	correct vaccinated	inflammatory/infectious disease	positive	640	1280
109	German Shepherd	male	intact	10	incomplete	tumour	negative	80	40
110	Border Collie	female	intact	15	correct vaccinated	neurologic disorder	positive	80	80
111	Boxer	female	intact	8	correct vaccinated	tumour	positive	40	10
112	Doberman	male	intact	4	unknown	cardiac disorder	positive	320	160
113	Jack Russel Terrier	female	intact	10	unknown	tumour	positive	320	160
114	Weimaraner	female	neutered	5	correct vaccinated	tumour	positive	640	320
115	Weimaraner	male	neutered	7	correct vaccinated	orthopedic disorder	positive	40	40
116	English Springer Spaniel	female	neutered	9	unknown	inflammatory/infectious disease	positive	10	40
117		female	neutered	4	correct vaccinated	healthy	positive	<10	40

118	mix	female	neutered	10	incomplete	healthy	positive	40	80
119	mix	female	neutered	11	incomplete	healthy	positive	160	320
120	mix	male	neutered	17	correct vaccinated	healthy	positive	160	160
121	mix	female	neutered	9	correct vaccinated	healthy	positive	80	80
122	Labrador	female	neutered	13	correct vaccinated	healthy	positive	40	160
123	Labrador	female	neutered	7	correct vaccinated	healthy	positive	<10	40
124	mix	female	neutered	12	incomplete	healthy	positive	80	80
125	Golden Retriever	female	neutered	14	incomplete	tumour	positive	80	80
126	Dachshund	male	intact	7	unknown	orthopedic disorder	positive	80	160
127	Australian Shepherd	male	neutered	11	unknown	tumour	positive	640	640
128	Doberman	male	neutered	12	unknown	tumour	positive	20	10
129	Newfoundland	female	intact	2	correct vaccinated	orthopedic disorder	positive	320	160
130	Doberman	female	intact	3	correct vaccinated	healthy	positive	20	20
131	German shepherd	female	neutered	7	correct vaccinated	inflammatory/infectious disease	positive	640	640
132	Dachshund	male	intact	5	unknown	inflammatory/infectious disease	positive	<10	<10
133	Labrador	male	intact	9	unknown	orthopedic disorder	positive	160	80
134	Dachshund	male	intact	12	unknown	various disorder	positive	<10	<10
135	Cavalier King Charles Spaniel	male	neutered	9	correct vaccinated	inflammatory/infectious disease	positive	20	<10
136	Foxhound	male	neutered	7	correct vaccinated	inflammatory/infectious disease	positive	40	80
137	Strobel	male	neutered	13	incomplete	tumour	negative	10	40

138	West Highland Terrier	female	neutered	16	incomplete	cardiac disorder	negative	160	80
139	mix	female	neutered	8	unknown	inflammatory/infectious disease	positive	40	80
140	Portuguese Podengo	female	neutered	15	correct vaccinated	inflammatory/infectious disease	positive	1280	640
141	mix	female	neutered	7	unknown	tumour	positive	640	320
142	Keeshond	male	intact	9	correct vaccinated	healthy	positive	320	160
143	Collie	female	intact	12	correct vaccinated	tumour	negative	160	80
144	mix	male	neutered	3	unknown	neurologic disorder	negative	1280	320
145	Belgian Shepherd	male	neutered	11	correct vaccinated	tumour	negative	20	40
146	Parson Russel Terrier	male	intact	6	correct vaccinated	neurologic disorder	negative	160	80
147	German Shorthair	female	neutered	14	correct vaccinated	cardiac disorder	negative	320	80
148	mix	female	neutered	16	incomplete	cardiac disorder	negative	<10	<10
149	Doberman	female	intact	2	unknown	cardiac disorder	negative	320	80
150	Beagle	male	intact	11	incomplete	cardiac disorder	negative	10	20
151	Small Münsterländer	female	intact	10	correct vaccinated	tumour	positive	1280	640
152	Bernese Mountain Dog	female	neutered	5	incomplete	inflammatory/infectious disease	negative	160	160
153	mix	male	neutered	9	correct vaccinated	various disorder	positive	320	160
154	Doberman	female	intact	6	correct vaccinated	cardiac disorder	positive	<10	<10
155	Australian Cattle Dog	male	intact	8	correct vaccinated	neurologic disorder	positive	20	20
156	Parson Russel Terrier	male	intact	12	incomplete	cardiac disorder	positive	40	160
157	Labrador	male	neutered	8	correct vaccinated	various disorder	positive	320	160

158	Bernese Mountain Dog	male	intact	7	correct vaccinated	tumour	positive	10	20
159	Black Russian Terrier	female	intact	10	unknown	inflammatory/infectious disease	positive	80	40
160	Beagle	female	neutered	5	correct vaccinated	various disorder	positive	160	160
161	mix	female	neutered	9	correct vaccinated	healthy	positive	320	160
162	French Bulldog	female	intact	2	correct vaccinated	inflammatory/infectious disease	positive	<10	<10
163	Doberman	male	neutered	7	correct vaccinated	cardiac disorder	negative	10	20
164	Small Münsterländer	male	neutered	13	correct vaccinated	endocrine disorder	positive	<10	<10
165	Doberman	female	neutered	6	correct vaccinated	neurologic disorder	negative	40	40
166	American Cocker Spaniel	male	intact	9	correct vaccinated	cardiac disorder	negative	40	80
167	Labrador	male	intact	8	unknown	tumour	negative	20	20
168	Golden Retriever	male	neutered	11	correct vaccinated	tumour	negative	160	40
169	mix	male	neutered	10	incomplete	inflammatory/infectious disease	negative	40	40
170	Labrador	female	neutered	10	unknown	neurologic disorder	negative	80	40
171	mix	female	neutered	9	correct vaccinated	healthy	negative	80	80
172	Black Russian Terrier	female	intact	6	correct vaccinated	endocrine disorder	negative	40	80
173	Border Collie	male	intact	3	correct vaccinated	neurologic disorder	negative	160	80
174	English Setter	male	neutered	2	correct vaccinated	orthopedic disorder	negative	160	160
175	German Shepherd	female	intact	9	correct vaccinated	orthopedic disorder	negative	80	40
176	mix	male	intact	15	correct vaccinated	neurologic disorder	negative	40	40

177	mix	female	neutered	16	correct vaccinated	inflammatory/infectious disease	negative	320	160
178	Labrador	male	neutered	6	correct vaccinated	inflammatory/infectious disease	negative	160	80
179	Australian Shepherd	female	neutered	10	correct vaccinated	cardiac disorder	negative	40	20
180	French Bulldog	female	intact	2	unknown	healthy	negative	160	160
181	Golden Retriever	female	neutered	14	correct vaccinated	endocrine disorder	positive	20	40
182	Hovawart	male	intact	4	correct vaccinated	inflammatory/infectious disease	positive	20	80
183	Husky	female	intact	12	unknown	tumour	negative	40	40
184	White Shepherd	female	neutered	8	correct vaccinated	orthopedic disorder	positive	160	80
185	mix	male	neutered	11	incomplete	various disorder	negative	320	80
186	Doberman	male	intact	1	correct vaccinated	orthopedic disorder	negative	40	160
187	Golden Retriever	male	neutered	11	incomplete	tumour	positive	160	160
188	Doberman	female	neutered	9	correct vaccinated	cardiac disorder	positive	20	10
189	Shetland Sheepdog	male	intact	7	correct vaccinated	inflammatory/infectious disease	positive	40	40
190	mix	female	neutered	12	incomplete	tumour	negative	320	160
191	Shih Tzu	female	intact	10	correct vaccinated	healthy	positive	640	1280
192	mix	female	neutered	3	incomplete	inflammatory/infectious disease	positive	640	640
193	Beagle	female	intact	3	correct vaccinated	inflammatory/infectious disease	negative	640	320
194	mix	male	intact	5	correct vaccinated	inflammatory/infectious disease	negative	160	80
195	Beagle	male	intact	5	unknown	healthy	positive	80	40
196	mix	female	neutered	12	correct vaccinated	orthopedic disorder	positive	160	80

197	Beagle	female	intact	9	unknown	healthy	negative	<10	40
198	mix	female	neutered	14	correct vaccinated	endocrine disorder	positive	320	320

[1] CAV, canine adenovirus
